# Transport-Layer Limitations for NFV Orchestration in Resource-Constrained Aerial Networks

**DOI:** 10.3390/s19235220

**Published:** 2019-11-28

**Authors:** Luis F. Gonzalez, Ivan Vidal, Francisco Valera, Borja Nogales, Victor Sanchez-Aguero, Diego R. Lopez

**Affiliations:** 1 Telematic Engineering Department, Universidad Carlos III de Madrid, Avda. Universidad, 30, 28911 Leganés (Madrid), Spain; ividal@it.uc3m.es (I.V.); fvalera@it.uc3m.es (F.V.); bdorado@pa.uc3m.es (B.N.); victor.sanchez@imdea.org (V.S.-A.); 2 IMDEA Networks Institute. Avda. del Mar Mediterráneo, 22, 28918 Madrid, Spain; 3 Telefónica I+D. C/Zurbarán, 12, 28010 Madrid, Spain; diego.r.lopez@telefonica.com

**Keywords:** NFV, Management and Orchestration (MANO), SUAV, intermittent availability

## Abstract

In this paper, we identify the main challenges and problems related with the management and orchestration of Virtualized Network Functions (VNFs) over aerial networks built with Small Unmanned Aerial Vehicles (SUAVs). Our analysis starts from a reference scenario, where several SUAVs are deployed over a delimited geographic area, and provide a mobile cloud environment that supports the deployment of functions and services using Network Functions Virtualization (NFV) technologies. After analyzing the main challenges to NFV orchestration in this reference scenario from a theoretical perspective, we undertake the study of one specific but relevant aspect following a practical perspective, i.e., the limitations of existing transport-layer solutions to support the dissemination of NFV management and orchestration information in the considered scenario. While in traditional cloud computing environments this traffic is delivered using TCP, our simulation results suggest that using this protocol over an aerial network of SUAVs presents certain limitations. Finally, based on the lessons learned from our practical analysis, the paper outlines different alternatives that could be followed to address these challenges.

## 1. Introduction

The future introduction of 5G technologies into the market is expected to completely change the telecommunications industry for the next decade to come: increased data rates, improved wireless transmission latency, and cost optimization will bring new and exciting opportunities to develop novel functionalities and applications for both consumers and the industry itself [1]. However, these objectives can only be reached with an appropriate evolution of our contemporary technologies, since current mechanisms may not provide a viable and cost-effective solution to meet the ever-increasing data demands. In this context, we can see why the Network Functions Virtualization (NFV) paradigm has been perceived by many researchers and operators as one of the key enablers for 5G, since the softwarization of network services and functions can reduce both deployment and maintenance costs, as well as easing its development and maintenance.

Another technology that has received significant attention in recent years has been Small Unmanned Aerial Vehicles (SUAV). Due to their limitless mobility and their capacity to onboard diverse hardware equipment as their payload, they provide a powerful tool that enables a wide range of new and appealing applications and services, such as road traffic monitoring using SUAVs [2], providing support for search and rescue missions [3] and natural disasters [4]. Moreover, given that they may be used to transport computing, networking and storage resources (e.g., by onboarding single-board computers), the research community has taken the first steps to build NFV infrastructures based on these SUAVs to support the cost-effective deployment of network services with data communications over delimited geographic areas [5].

However, despite the existing research efforts, there still are important challenges and hurdles that need to be addressed to effectively support NFV operations over resource-constrained platforms, such as those that can be onboarded on SUAVs. In an exploratory research work [6], we succinctly identified these hurdles. In this paper, we continue this research line, presenting a more detailed analysis of these challenges from a theoretical perspective. Following this analysis, the paper focuses on a specific but relevant aspect: studying how the intermittent availability of the aerial communications affect the dissemination of NFV orchestration information, i.e., the information that has to be exchanged to coordinate the computing, networking and storage resources onboarded on SUAV units. In particular, the paper presents the results of several simulation scenarios, using both TCP (the transport protocol commonly used to disseminate orchestration information in traditional cloud computing environments) and UDP, in order to measure the performance of orchestration traffic in an aerial network composed of several SUAVs. Moreover, we analyze the behavior of both transport protocols in idle scenarios (i.e., no presence of background traffic sharing the wireless medium with the orchestration traffic) and in scenarios with congested wireless links. Our simulation results suggest that the use of TCP to disseminate orchestration information across an aerial network of SUAVs presents some limitations. Considering these results, the paper anticipates a set of research directions for contributing to a future view of an effective integration of NFV technologies into SUAV platforms.

The rest of this paper is structured as follows: in Section 2, we look at NFV concepts, including NFV orchestration and its main principles, and we review the existing literature regarding SUAVs and some of their applications. In Section 3, we introduce NFV orchestration in aerial ad-hoc networks, analyzing the potential challenges and hurdles of the use of NFV technologies in this new environment, and pointing out potential approaches that could be followed to address those challenges. In Section 4, we perform a set of simulations to test how TCP and UDP traffic performs in an intermittently available SUAV network scenario with different characteristics, analyzing the obtained results. Finally, Section 5 presents the main conclusions and future directions of our work.

## 2. Background and Related Work

Improving data rates and lowering communication latencies are some of the main challenges for developing new infrastructures and technologies for the 5th generation of mobile networks in the near future. However, accomplishing these objectives requires evolving and adapting all current technologies and concepts to produce significant improvements in those areas, by introducing new paradigms with the potential to reshape the next decade of mobile infrastructures. In this fashion, NFV has risen as one of the key enablers of 5G communications, aiming at softwarizing currently deployed physical network functions, i.e., eliminating the usage of specialized equipment to build network functions in favor of using generic hardware.

The main guidelines for creating NFV deployments were defined by the NFV Industry Specification group through a reference architectural framework described in [7]. In this document, Virtualized Network Functions (VNFs) are considered the central elements of the architecture, defined as software implementations of network functionalities (e.g., a router, a load balancer or a traffic generator). These elements are deployed in an NFV Infrastructure (NFVI), which has all the required storage, computing and networking resources needed to support the virtualization and execution of network functionalities. Moreover, the NFVI elements (nodes) provide an abstraction layer to separate VNF functionality from the hardware being used underneath, ensuring that no further specialized equipment is necessary unless a VNF specifically demands it. This behavior saves costs in both network deployment and maintenance, as well as simplifying new network functionalities development.

However, having isolated VNFs (i.e., network functionalities) is not enough to establish a complex environment for providing end-to-end services such as those present in currently traditional networks. In this case, VNFs interconnect between themselves to generate Network Services (NSes), whose purpose is building more complex behaviors while easing the service chain provisioning for telecommunication operators and Service Providers.

To help coordinating operations over the NFV environment, the same NFV Industry Specification group defined a Management and Orchestration (MANO) system. This component is responsible for deploying and managing the lifecycle of VNFs inside the NFV infrastructure. To cover all lifecycle requirements and phases, the MANO system is divided into three different components: (1) the Virtualized Infrastructure Manager (VIM), responsible for controlling and monitoring the resources of an NFV infrastructure (including storage, networking and computing resources); (2) the Virtual Network Function Manager (VNFM), responsible for instantiatiating, configuring, modifying and terminating VNFs; and (3) the NFV Orchestrator (NFVO), in charge of coordinating the deployment of individual VNFs, and their interconnection, to build the requested service graph.

On the other hand, SUAVs have been an attractive technology for the research community thanks to their increased mobility support with respect to other technologies, such as terrestrial vehicles. There has been quite an effort to develop new applications using these devices in many engineering fields. Examples of these applications are supporting search and rescue operations [3], forest fire automatic detection [4], precision agriculture [8] or moving cargo from different warehouses along a delimited geographical area [9], just to name a few of them. SUAVs have also been considered to provide networking functionalities and services over geographic areas. The work in [10] presents a solution that uses UAVs for supporting backbone communications to mobile ground stations. In [11], the authors design a solution to enable a broadband network for emergency communications using geolocalization. The work in [12] presents several architectures for creating a computing platform based on several aircraft, discussing the possibility of using UAVs for data collection in wireless sensor networks as well. In [13], a UAV network architecture is presented to relay data traffic for ground vehicles, which also supports data collection functionalities.

However, we want to highlight that according to our previous research on this area, there is still a lack of consensus from the research community and the industry on a standard design for SUAVs that using existing protocols and technologies, enables the flexible execution of general-purpose telecommunication services and functionalities [14]. Despite the market opportunities in this area, commercial SUAV products are typically proprietary solutions, i.e., usually manufactured to accomplish specific missions.

In our future view, swarms combining UAVs of different sizes and characteristics might be positioned over delimited geographic areas, and provide a baseline resource infrastructure, capable of flexibly executing different network functions and services, which could then be offered on-demand by a service provider over that areas. As an example, a telecommunication operator could deploy several SUAVs to temporarily provide Internet access over a specific geographic location, for instance because the access network infrastructure of the telecommunication operator in that area is unavailable (e.g., as a consequence of a natural disaster) or insufficient (e.g., in a crowded event). In these cases, SUAV platforms could enable a cost-effective deployment of network functionalities (e.g., wireless access points, routing functions, IP telephony servers, etc.) over a target geographic area. This way, SUAVs could be used to complement the available network infrastructure over specific areas, which could favor the provision of the network services expected for 5G.

To realize this view, NFV emerge as an enabling technology to decouple network functions and services from the hardware they are deployed on: by using virtualization, network services can be executed independently of the hardware and software platforms used underneath. This would allow their execution on multiple and heterogeneous aircraft and devices, as long as these can support virtualization. This way, NFV has the potential to take fleets of SUAVs to a higher degree of flexibility in the provision of network functions and services. A degree of flexibility that can barely be achieved with other specialized SUAV equipment that has been designed and/or configured offline to provide specific services. In addition to its inherent flexibility to adapt the SUAV functionalities to serve to different purposes, the use of NFV brings other relevant advantages in the field of the SUAV arena, particularly: simplifying the development cycle of functions and services, which may prototyped and tested in production-like virtualized environments; easing the evolution of these functions and their migration to production state, as they are implemented in software; and the facility to adapt the resources allocated to specific functions and services to better accommodate varying demands (e.g., an increasing number of users).

In this respect, the research community is giving the first steps towards realizing the softwarization of network functions in aerial networks. Examples of this approach can be seen in works such as [15] where authors use Software-Defined Networking (SDN) techniques to manage a UAV network, [16] where authors provide efficient handover techniques using SDN, with the aircrafts being used as OpenFlow switches, or [17] where authors use SDN in aircraft to improve connectivity between aircraft of an aerial network. In [18], the authors explore the use of SUAVs as network nodes, with the capacity to extend the programmable infrastructure of the 5G network; these SUAVs are able to onboard all the required computing, storage and network resources to provide a fully working NFVI platform where VNFs can be deployed, configured and run, saving on infrastructure deployment and maintenance costs in comparison with traditional NFVIs. This concept is further developed in [5,19], where the authors introduce a new NFV system design able to support the agile configuration and deployment of moderately complex NSes over a cloud platform composed of SUAVs with constrained equipment and resources. On the other hand, The NFV-SUAV combination has delivered of its benefits can be seen in [20], where authors propose a full video-surveillance system for big poorly internet-covered areas using UAVs, whose mobility can be used to distribute the aircraft along an specific geographical region to obtain video footage, using NFV as the platform to transmit the video signal through a network of several VNFs running inside the aircraft. This behavior can be considered rather complex to perform through pure virtualization, due to the execution times needed for performing all aircraft actions, which might not be fast enough to provide the service, especially since the amount of resources that can be onboarded are constrained by their space and weight to allow the aircraft to stay in the air as much as possible. Instead, authors propose using paravirtualization, where Virtual Machines (VMs) share the hardware directly with their host, allowing its Operative System (OS) to be a platform for directly (and exclusively) operating with the VNFM, saving valuable execution time and allowing a better management of the resources on the aircraft. Other outstanding work line in this area is related with the migration of VNFs between the NFV units of a network composed of several SUAVs in order to perform a seamless transition of VNFs between UAVs to minimize service cuts and/or reduce the amount of time a service remains offline. This behavior can only be performed if all the associated network services, routing and operational control migrate quickly. An example of this can be seen in [21], where authors propose an NFV-based solution that takes into account the high-mobility requirements of these networks. The work in [22] presents an architecture of function softwarization in an irrigation system based on a network of sensors and UAVs.

## 3. Orchestration in Intermittently Available Platforms

As we have described in the previous section, the research community has shown growing interest towards the softwarization of network functions over SUAV platforms: these aircraft may have enough potential to provide a flexible environment to run VNFs on an NFVI infrastructure, allowing development and creation of a wide variety of NSes. Furthermore, this infrastructure can take advantage of its mobility to deploy these NSes in any kind of environment/area where common vehicles might not be able to access or have difficulties to do so, e.g., natural emergencies, or would imply additional costs on deploying and managing a complete infrastructure, e.g., remote rural areas with no easy access. Moreover, some VNFs could also take advantage of the mobility of the aircraft to build NSes that require movement to perform any set of actions (e.g., moving cargo from different warehouses with predefined paths over a delimited geographical area [9] or a road traffic monitoring system [2]).

**Figure 1:**
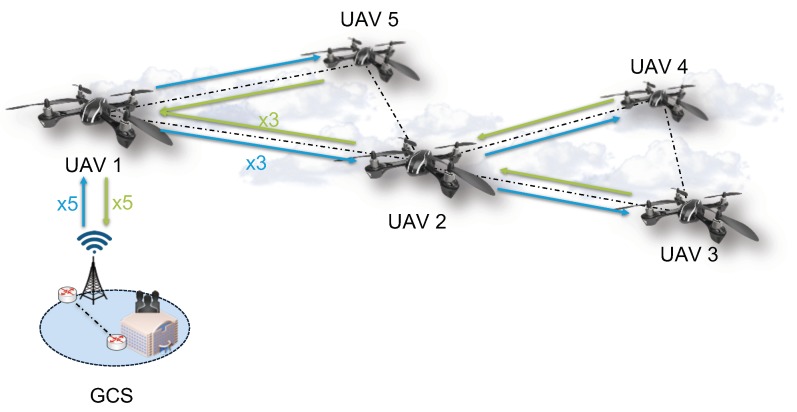
Example of a NFV-SUAV network with their corresponding HTTP orchestration traffic flows.

However, a prior work of the authors [6] points out the existence of several challenges that should be taken into account when dealing with NFV in SUAV scenarios, to enable the automatic deployment, configuration, and operation of the aforementioned NSes. In the following, we provide a more comprehensive description of these hurdles, using the reference scenario shown in Figure 1 as an example.

As it can be seen in Figure 1, the scenario is composed of several SUAVs with enough computational, storage and networking resources to build an NFV infrastructure, i.e., each one of the aircraft is an NFVI node or serves to onboard an NFVI node as payload. These NFVI nodes are interconnected through wireless communication technologies (e.g., WiFi of line-of-sight radio links), building a Flying Ad-Hoc Network (FANET) that enables multi-hop data communications. The infrastructure is controlled from a Ground Control Station (GCS), where a VIM is located. The NFVO will communicate with the VIM to coordinate the automatic deployment of NSes over the ad-hoc network formed by the NFVI nodes, as well as retrieving information from the status of the compute, storage and network resources, as well as from the VNFs, from each one of the aircraft. Every aircraft can be placed at any location over a delimited geographical area, either remaining its position static the whole time or moving through a scenario, both autonomously or instructed by an operator from the GCS, depending on the situation and the type of aircraft and/or service.

Despite all its parallelisms with traditional cloud-based NFV infrastructures, there are some differences that must be pointed out. First, SUAVs have a communication range between aircraft due to the limitations of wireless communications. In consequence, they need an adequate distribution to provide enough coverage for the whole target area, implying that not all SUAVs will be able to reach the GCS directly in most cases and, in consequence, VIM-SUAV communication will be mostly performed through intermediate nodes, forwarding both orchestration traffic (i.e., the traffic needed to manage and monitor the available NFVI resources and/or services) and data traffic (information transmitted and/or received by VNFs as part of their normal operation) through the network. Finally, when mobility is enabled, SUAVs may be constantly breaking links that might disrupt SUAVs communication, as new ones must be dynamically created again with other nodes constantly, which can harm certain types of communications. Moreover, these SUAVs need the aid of a battery to stay afloat in the air during an operation/service, requiring a subsequent replacement when this battery is depleted, disrupting even more this communication.

Despite all those differences, in a prior work [5], we presented a functional prototype of an NFV system capable of deploying virtual functions and services over resource-constrained SUAV networks. This work served to demonstrate that the introduction of NFV components and processes does not prevent nor negatively impacts the proper operation of the system. Regarding mission planning functionalities, they can be delegated to external modules in charge of specifying the NFV descriptors of the network services to be deployed, in addition to other parameters such as those related to the configuration of the VNFs.

In another previous work [6], we aimed at identifying all these hurdles that could be present when dealing with NFV orchestration in SUAV platforms, classifying them into different categories as well as proposing some alternatives to solve, or at least mitigate, these difficulties in future implementations. In the following subsections, we use that same classification as well in order to analyze these aspects and, in later sections, we test the impact of several of these challenges on NFV orchestration in FANET environments, using a simulation platform.

### 3.1. Limited Lifetime of NFVI Nodes

SUAVs are autonomous devices that require some power supply source to be operative. As these units spend most of their time flying, they usually incorporate an autonomous rechargeable battery to act as its power source. Naturally, performing different actions (e.g., routing, transmitting/receiving wireless traffic, executing software functions, etc.) will eventually deplete their battery lifetime: even when they are not executing any specific task, the mere act of staying in the air will reduce their charge until their eventual depletion. Hence, all VNFs running inside its payload must be migrated into another operative unit to avoid, or mitigate, operational cuts on the services being provided. Unfortunately, this action is not as trivial as it may seem at first: not only exists several limitations regarding the computing, storage and networking resources involved in the migration, but also its transition should be anticipated with enough time to ensure a seamless migration procedure, only possible when a VIM is able to monitor the battery lifetime of the aircraft. This VIM could use algorithms to indicate when the aircraft should leave/enter a recharging station such as the one seen in work [23] or [24], allowing it to also predict when the services would need migration before replacing the unit. However, at the time this paper is being written, most VIM implementations do not consider battery lifetime as a limited resource, making VNF migration in these environments suboptimal.

Other measures to preserve battery lifetime could be perching some SUAVs on land, for instance in specific-purpose ground structures, or using different batteries for flight management and computational resources (making sure that the battery on the payload exceeds the flight time of the other one).

### 3.2. Intermittent Availability of Control Communications

As we have previously mentioned, SUAVs battery depletion turns these units into volatile nodes that must be replaced by other units. These disconnections will break links between the SUAVs because they are organized in a multi-hop wireless ad-hoc network, causing communication interruptions between nodes and the GCS. This disruption will be mostly transient however, as either the routing protocols will likely find a new path, and/or the units are replaced after some time have passed to converge into a stable state. Unfortunately, during this period, the units affected may be momentarily unavailable to the MANO system, which will not be able to either deploy or configure VNFs in them, even though those resources will be working properly with their VNFs running normally. Hence, it is important for a VIM to consider that this transient state is the normal behavior on the aircraft, and not interpret these failures as permanent. One possible solution could be supporting a reasonably increased delay when performing orchestration and/or expecting information from the NFVI nodes inside the network. Another possible alternative to support the increased delay could be using new paradigms such as Delay Tolerant Networking (DTN), whose main characteristics consists of storing its data until there is an available hop to reach the next node to the destination (i.e., it is not necessary to have an available path, but rather finding available nodes and forward the traffic until it reaches its destination). Nevertheless, the VIM should adapt to the increased delay on the traffic caused by the network as well, providing a higher window to receive/send updates of the state of the NFVI resources on the devices.

Moreover, battery depletion is not the only phenomena that can produce these communication failures. SUAVs rely on wireless media for their information exchange, which is a far less reliable medium because it can be affected by a lot of parameters involved during the communication, e.g., weather conditions, distance, frequency used, etc., introducing more link breakdowns and eventual reconnections. This problem can be amplified when mobility is added to the equation, as topology changes and aircraft temporarily being out of range will induce more of those disconnections, causing the temporary unavailability of NFVI nodes in consequence.

### 3.3. Limited Capacity of NFVI Nodes

It is important to emphasize that SUAVs are smaller than regular aircraft. In consequence, their payloads must be reduced in weight and size to be onboarded without harming their flight capabilities. This impacts the nature and the capacity of the available resources in every NFVI unit provided by the SUAVs. This issue has been explained in more detail in [5], where authors encourage the use of lightweight VNFs with container virtualization instead of the traditional hypervisor-based one to overcome this resource limitation. An alternative to these lightweight containers could be the one seen in [20], where authors propose using paravirtualization instead. This technique allows a VM to directly communicate with the OS directly, as opposed to hypervisor virtualization where the VM communicates with its “virtualized kernel”. This direct communication speeds the information exchange between the host and the VM (or in this case VNF), increasing orchestration efficiency in the process.

SUAVs limitations are not exclusive to the equipment being used on their payloads: how VIMs exchange information with NFVI units also plays an important role in their communication performance. Most commercial VIMs, which are highly based on OpenStack [25], use HTTP as their default application-layer protocol for exchanging information related with their NFVI resources, getting information about the status of the resources and/or sending actions to be applied on both resources and/or VNFs. With this context in mind, it is understandable the selection of this protocol for this purpose: not only is one of the most used application-layer protocol on the Internet, but also the HTTP embedded Representational State Transfer (REST) model perfectly fits with its information exchange that has to be performed between the aircraft and the GCS. However, this protocol was never intended to operate as a lightweight protocol, as it can be considered a process-intensive protocol compared to alternatives such as the Constrained Application Protocol (CoAP) [26] or Message Queuing Transport (MQTT) [27]. This could be a problem in SUAV networks, since the resources that can be onboarded cannot be very powerful due to both its limited size to fit equipment and the necessity of saving precious battery lifetime. Therefore, some of the aforementioned protocols could be used as alternatives to reduce message overhead for a more cost-effective solution to save battery lifetime and computational resources needed to process the messages.

### 3.4. Transport-Layer Protocols for Control Communications

Continuing with the VIM-SUAVs communication, we must focus on certain aspects related with the behavior of HTTP that harms the overall performance of NFV orchestration in SUAV scenarios. HTTP uses TCP as its default transport-layer protocol. Although this protocol is one of the main pillars of telecommunications in current networks, certain works have shown that TCP performance over (mobile) ad-hoc networks makes it not the most appropriate choice for them, as it can be severely lowered compared to completely wired networks [28,29,30,31,32]. On its conception, TCP was developed as a protocol for reliable data transfer over wired networks. In most cases, link-related error would rarely occur, and most packet losses would be related to congestion. However, wireless technologies completely changed how communications could be done using more than cables to interconnect devices, allowing free node movement as well as flexibility to the distribution of a network, but also introduced new challenges that increased link-related failures due to several factors such as the instability of the medium and mobility. In consequence, TCP was never intended to deal with temporary and/or common link failures, which could potentially affect its use in wireless environments, creating new drawbacks that had to be dealt with in order to ensure efficient communication through this medium. Most of them can be found in [28,29], but the most relevant for NFV orchestration in SUAV networks are the following ones:

- Physical layer: Wireless links induce more errors in the packets. Problems such as fade-away and interferences can modify bits during the transmission, while poor reception/channels will create packet losses, severely impacting its performance [28]. The reason for this decrement is a constant retransmission of packets due to these losses/modifications, keeping its congestion window low and reducing the overall throughput in consequence [29].
- Network layer: Routing in ad-hoc networks has some differences over traditional networks, using protocols such as Dynamic Source Routing (DSR) [33], Ad-hoc On-Demand Distance Vector (AODV) [34] or Optimized Link-State Routing Protocol [35]. These protocols assume that nodes form dynamic topologies that may change over time. Therefore, they need some time to recompute their routes when one or more nodes become unavailable before reaching a stable state, which can be dependent on parameters such as the chosen mobility model [30]. However, the disparity between the time to recompute a route and TCP retransmission timers may cause the sender to use outdated routes, increasing the number of retransmissions performed and, again, reducing the congestion window along with its throughput. This problem is further exacerbated when the number of intermediate nodes is increased, as they must also recompute routes as well [31].

Most of the problems described above can be summarized in one sentence: TCP is not able to recognize the source of the failure in wireless media. Therefore, it invokes the only mechanism to, in theory, increase overall transmission performance: the congestion control mechanism. However, here it is disadvantageous because, in almost all cases, there is no congestion in the network, but the protocol interprets SUAV disconnections as a congestion problem even though it is not the case. Hence, this reaction will decrease throughput, harming overall performance instead of increasing it since there is no congestion in the network, as the control information is usually not high in comparison with other types of data exchange between nodes.

Although TCP might not be the most suitable option to transmit information in SUAV environments, we still want some of its characteristics when transmitting control information in a NFV context, as it is vital to ensure reliable data transfer to every node inside the network. However, we also need to avoid harming its throughput and performance as much as possible for control communications, which is the main problem that TCP can introduce in these environments. One possible alternative to solve it could be using protocols that rely on UDP as its main transport-layer protocol, moving its reliability mechanisms to the application layer since this information should safely arrive to the aircraft. In this fashion we can find CoAP, which tries to apply the HTTP REST model to constrained environments but reducing its overhead size as well as using UDP as its transport-layer protocol. Similarly, another attractive protocol that uses UDP is QUIC [36], which tries to mimic an implementation of HTTP (version 2 [37]) and Transport-Layer Security (TLS) [38]. QUIC would allow keeping the REST model intact used for the exchange of information between the VIM and the aerial nodes. In any case, we want to highlight that QUIC is still a connection-oriented transport-layer protocol, which implements a congestion control mechanism based on that of TCP. Therefore, it may be sensitive to the same limitations that can be observed for TCP.

### 3.5. Enhanced Policies for VNF Placement

Recalling how VIM work in NFV orchestration, they usually take into account certain parameters for allocating VNFs on the NFVI nodes (e.g., CPU, memory usage, etc.). However, battery life is not included among those parameters, so VIMs do not take it into account when deploying/managing VNFs on NFVI nodes. Nonetheless, if a VIM were able to use this parameter in SUAV NFV environments, SUAVs could potentially improve its energy efficiency by assigning critical VNFs to healthier nodes, i.e., those aircraft with extended battery lifetime. Moreover, orchestration could also be improved by using this parameter, as knowing the remaining capacity is essential to trigger VNF migration (discussed in more detail in Section 3.1) and support re-allocation policies.

Another aspect that needs to be addressed is the placement of SUAVs over an area. In traditional scenarios, NFVI node placement might not be as critical, as these nodes are placed in fixed locations interconnected through a cabled network. In this case, however, SUAVs can move around a delimited field, but its target position for their trajectories should be provided to the flight control engine running at the SUAV. The authors in [19] propose implementation of the flight control engine as a VNF on every unit, providing its flight trajectories using a VNFM for providing its flight trajectories as configuration parameters. Applying this method, these trajectories could be specified in the deployment to the NFVO, or even appear in the NS descriptor to be configured on the NS deployment.

### 3.6. Challenge Summary

In this subsection, we aim to summarize all of the challenges and hurdles present in resource-constrained aerial networks in Table 1, introducing the main problems of each category as well as some alternatives that could provide a solution to them.

## 4. Practical Evaluation of Transport-Layer Options

Previously, we have described the potential challenges and hurdles in NFV orchestration when dealing with intermittently available SUAV platforms. In this section, we analyze a subset of these problems from a practical perspective, performing different tests to gain a deeper understanding of their impact. In particular, we break down the consequences of using TCP as the default transport-layer protocol to support the exchange of orchestration information over SUAV networks, since some of its mechanisms could prove to be problematic in these scenarios.

As mentioned in the previous section, commercial VIMs mostly use HTTP to exchange orchestration information with the compute nodes under their control. Consequently, these communications are typically established using TCP. Although this can be a good solution to support management and orchestration communications in traditional scenarios such as datacenters, where fixed network technologies at Gb/s speeds may be available, SUAV platforms behavior turns some of the principal characteristics of TCP into mechanisms that may harm the performance of orchestration traffic, instead of helping its correct delivery. One example of this is related with the congestion control mechanism of TCP in FANET environments. Typically, the operation of VIMs requires low bit-rate traffic flows to manage and monitor the available NFVI resources and the VNFs at the SUAVs. These flows should appropriately be delivered given the bandwidth that may typically be present in wireless ad-hoc networks [5]. Theoretically, TCP should not decrease its traffic rate if the network has no congestion, since that will be ineffective in those cases. However, the presence of constant link breakdowns, due to both wireless link issues and SUAV replacements, can produce excessive TCP retransmissions or, in worse cases, sever the network path between the GCS and a SUAV completely, if an intermediate wireless link that has failed does not recover in a reasonable period of time. Moreover, given its importance to effectively coordinate the available NFV resources, we may want to prioritize the delivery of management and orchestration information against other kinds of traffic, as long as it does not monopolize the available bandwidth for data communications. This last point is fundamental, since achieving a timely dissemination of orchestration information is essential to efficiently manage the NFV resources present in the aircraft.

Having analyzed from a theoretical perspective how using TCP connections may affect management and orchestration operations in SUAV environments, we address a more detailed evaluation of this impact using a simulation platform. In particular, we use the well-known Network Simulator 3 (NS3), since it is flexible enough to simulate a target scenario with multiple SUAVS, while at the same time enables fundamental features such as SUAV replacements, the establishment of simultaneous TCP and UDP communications, and the provision of detailed traffic captures for their subsequent analysis [39].

**Figure 2:**
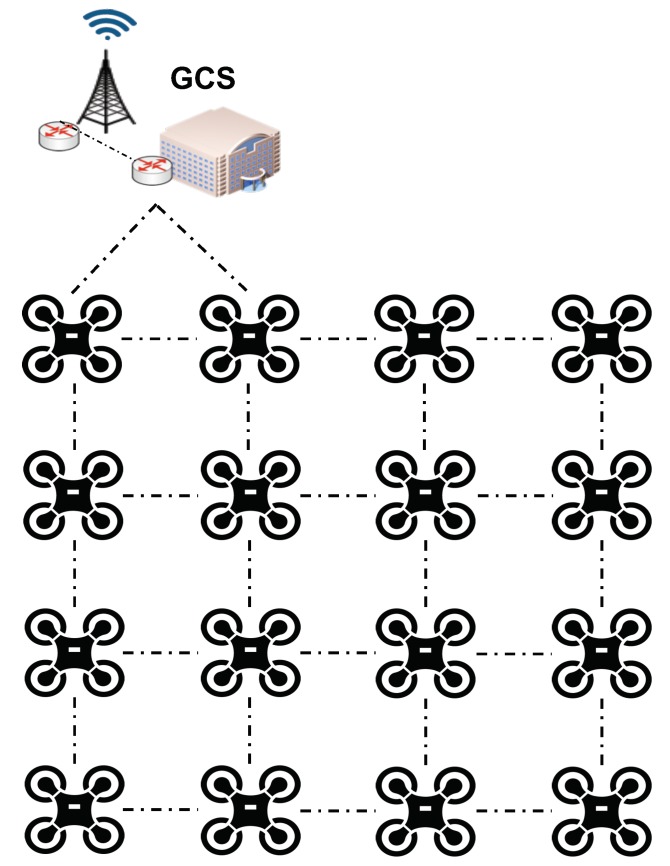
16 SUAV FANET scenario forming a grid topology. SUAVs (1,1) and (1,2) are directly connected to the GCS.

Figure 2 shows the considered reference scenario at the evaluation. We want to highlight that in a prior work [5], we developed a functional prototype of an NFV system capable of deploying virtual functions and services over resource-constrained SUAV networks. The results of that research work, provided the quantitative proofs for the assumptions made in this practical evaluation. The reference scenario comprises 16 SUAVs, interconnected in a 4 × 4 grid topology, and a GCS with two points of access to the aerial network conformed by the SUAVs. The chosen topology enables study of the performance of management and orchestration operations in a favorable environment, where multiple network paths exist between the GCS and each SUAV for the exchange of control information (i.e., there is no single point of failure). It is expected that other network topologies, where the degree of the aerial nodes (i.e., the number of wireless links of node to other neighboring nodes) is reduced, will produce worse performance results. Moreover, the number of aircrafts in the reference scenario has been considered sufficiently large to obtain trustworthy conclusions. In the scenario, we assume there is only a single VIM attached to the GCS, simulating the generation/reception of NFV management traffic to/from the SUAVs using a single MANO pile (i.e., all SUAVs NFVI belong to the same orchestration domain).

In our scenario, all aircrafts are provided with a single WiFi interface using IEEE 802.11a, which is configured to create an ad-hoc network with the former topology. In this topology, the GCS can communicate with the SUAV grid through two independent SUAV units. With this configuration, each SUAV and the GCS can communicate with other SUAVs in their vicinity using a single wireless network card. Multi-hop communications over the ad-hoc network built by the SUAVs and the GCS are guaranteed through the use of a network routing protocol. In particular, in our simulations OLSR for this purpose. We want to mention that according to our previous work [5], this approach, based on the use of a WiFi ad-hoc network and a network routing protocol, has proven to be effective to support multi-hop wireless communications in SUAV scenarios. However, this is not the only valid approach to guarantee end-to-end communications, as these could also be performed using alternative approaches, such as cellular device-to-device communications [40]. However, verifying the feasibility of this technology in our reference scenario would still require a detailed analysis, taking into consideration different aspects related to energy consumption, as well as size and weight of the enabling equipment.

In our evaluation, we consider the management and orchestration information that is exchanged between the GCS and each SUAV to monitor the status of the computing, networking, and storage resources onboarded on the SUAV. This type of control information is continuously transmitted in NFV scenarios, with the VIM periodically triggering each compute node to get updated information on the status of the resources. Our future work will consider other forms of management and orchestration traffic, such as the traffic needed to support the instantiation and deletion of VNFs over the SUAVs.

In typical production-like NFV environments, management and orchestration information is usually exchanged using the HTTP protocol, where the VIM can obtain information regarding the state of the resources at the compute nodes (in our case, the state of the resources onboarded by the aircraft). Because this protocol uses a REST model, aircraft will reply with the requested information to answer the query. This behavior is not required in the tests that we have performed however, since we are concerned on studying the overall orchestration traffic throughput on either direction (i.e., from the GCS to each SUAV, and vice versa), rather than precisely emulating the orchestration traffic pattern. Therefore, in our simulations we decoupled the bidirectional exchange of management and orchestration information between the GCS and each SUAV, representing it as two different Constant Bit-Rate (CBR) traffic flows. These CBR flows serve to approximate the periodic exchange of control information in typical VIM-NFVI communications. The transmission rate of each CBR source in our simulations was set to 7 kbps for each SUAV-to-GCS communication, and 5 kbps for the reverse path. Both values were selected to fit the average throughput that is seen in each direction when using a real VIM such as OpenStack, as shown in [5], where authors build a NFV scenario using real SUAV units and OpenStack as its VIM (located in a GCS). Following this approach, we were able to obtain results using realistic values for the transmission rates in this kind of control transactions, making it easier to perceive traffic variations and its evolution during the tests, and performing better comparisons with real scenarios. 

In addition to the previous points, we need to take into account that we are dealing with intermittently available units. Their most prominent source for their disruptions is the battery lifetime: every UAV in the aerial network must have a rechargeable battery to power it. To simulate this functioning, we model the battery lifetime using a uniform random variable with upper limit 21 min and lower limit 19 min, U (19,21), for a 20 min average lifetime. These have been identified as regular operational values of commercial off-the-shelf SUAV units (e.g., [41]). Since there are no additional battery drain sources that may reduce its battery lifetime because the nodes are static, its position will not affect its battery consumption as demonstrated in [42], which makes a uniform variable an appropriate choice for modeling the battery lifetime, keeping the SUAV consumption values stable throughout the simulations. In addition, every simulation starts with a battery offset for each SUAV, to take into consideration that in a real-case scenario, SUAVs could have been running for a period of time when they are deployed. Moreover, since we are using a battery, aircraft must be replaced when its battery has been completely depleted. This replacement might be instantaneous in an ideal scenario since a battery-monitoring VIM could use a specialized algorithm to plan ahead and schedule a replacement when the other aircraft starts is near its battery lifetime limit point. However, there could be many problems in the process (bad weather, planning errors, etc.). Hence, we opted for an exponential random variable with average 2 min, E (2), to model their corresponding time replacements to compensate for those uncertainties. With both models, we can simulate a battery depletion-replacement under more realistic conditions. In our reference scenario, we assume that every SUAV has an analogous aircraft in the GCS whose battery is completely recharged when the SUAV flying has been completely discharged.

We divided out tests into three different sets. In the first set, we aim at understanding how the exchange of orchestration information is affected by the intermittent availability of the SUAVs building the aerial ad-hoc network, through the analysis of its throughput. In a second set, we analyze how this information is affected by the presence of background data traffic among other nodes of the network (i.e., data-plane traffic corresponding to the network services deployed over the aerial network of SUAVs). In the first two sets, we assume the use of TCP to support the exchange of management and orchestration information between the GCS and each SUAV node. In a third set, we explore how UDP would behave in the previous scenarios and discuss the potential advantages that might be obtained when using UDP to deliver orchestration information in our reference scenario.

The TCP variant used for all the tests has been TCP NewReno [43], which provides a well-known reference congestion control mechanism. Finally, We chose OLSR [35] because it is a well-known and recognized standard in MANET environments, due to its simplicity and efficiency, it is supported by NS3 and also has open-source implementations (which will be used in our future work). Moreover, in a previous work [5] we demonstrated that OLSR is an appropriate protocol to support data dissemination over multi-hop flying ad-hoc networks where the position of the flying nodes is relatively static (that is, changes in the network topology are mainly caused by the replacement of nodes due to battery constraints).

In following subsections, we describe in more detail all the performed tests, as well as the observed results for each one of them. The parameters of the simulations, along with their corresponding set of values, are described in Table 2.

### 4.1. Orchestration Traffic Performance Using TCP

For this first test, we aim at checking orchestration traffic behavior when using TCP under constant link breakdowns due to battery depletions. As we have previously mentioned, the simulation will use two CBR sources with 5 kb/s to represent the GCS-to-SUAV communications and 7 kb/s for the UAV-to-GCS communication. Under normal conditions, where SUAVs do not require a battery, i.e., they are considered fixed nodes (left on the ground connected to the electrical network, for example) these rates are stable thought all SUAV communications except, for minor losses due to medium itself (neglectable loses). However, once sporadic link breakdowns become part of the equation, throughput is negatively affected when TCP is the transport-layer protocol, due to its congestion avoidance mechanism. Even though this functionality does prevent the source from sending more traffic to the network, it will affect the connection health since the links will never be congested with these type of orchestration traffic because the amount of traffic generated from these sources is very small compared to the available bandwidth. However, several temporary link breakdowns will affect those links because the sources will generate excessive retransmissions (which are unnecessary in this context since the problem is temporal). Moreover, using OLSR as our default routing protocol will require several route recalculations every time a node has to be replaced. This will impact all communications being forwarded on that node, and the relatively long update times (every 10 s an update message is broadcasted to its neighbors) might leave some aerial nodes without a network path with the GGS and, in consequence, will prevent the aircraft from receiving any control information for a certain amount of time, and the GCS of retrieving information regarding the status of those nodes. This is not a problem originated at the transport level, and it would still be present even if we used an alternative protocol to substitute TCP, e.g., UDP, because we will still need to wait until the routing protocol converges again to find an available route. However, this route convergence time will trigger the congestion avoidance mechanisms; because TCP was created without wireless technologies in mind, all link breakdowns will be interpreted as congestion failures regardless of its true origin, since those other types of failures such as host/link teardown are much less present in completely wired networks. Therefore, TCP will start to retransmit segments using its backoff timer by scheduling subsequent retransmissions to ensure that all information is sent to the other end while leaving some space for the network to recover from the (non-existent) congestion issue. This behavior could turn into a problem in FANET environments for several reasons: while the network is still recovering when a SUAV is being replaced, packets will use outdated routes to reach its destinations, adding unnecessary retransmissions that will slightly increase the overall traffic inside the network; most importantly, if a node cannot find a route before the backoff timers expire, either because of a network related affair or because the routing protocol was not fast enough to find a route, then the TCP connection will be completely closed by the sending party, potentially leaving a SUAV without any control information until it is replaced once again, i.e., when it opens a new connection with the GCS. This last problem could be fairly common in such scenarios, since some of the UAVs can be substituted simultaneously and have relatively long replacement times in certain situations, potentially leaving some aircraft without orchestration information for a long period of time, which could be avoided if another transport-level protocol is used instead.

**Figure 3:**
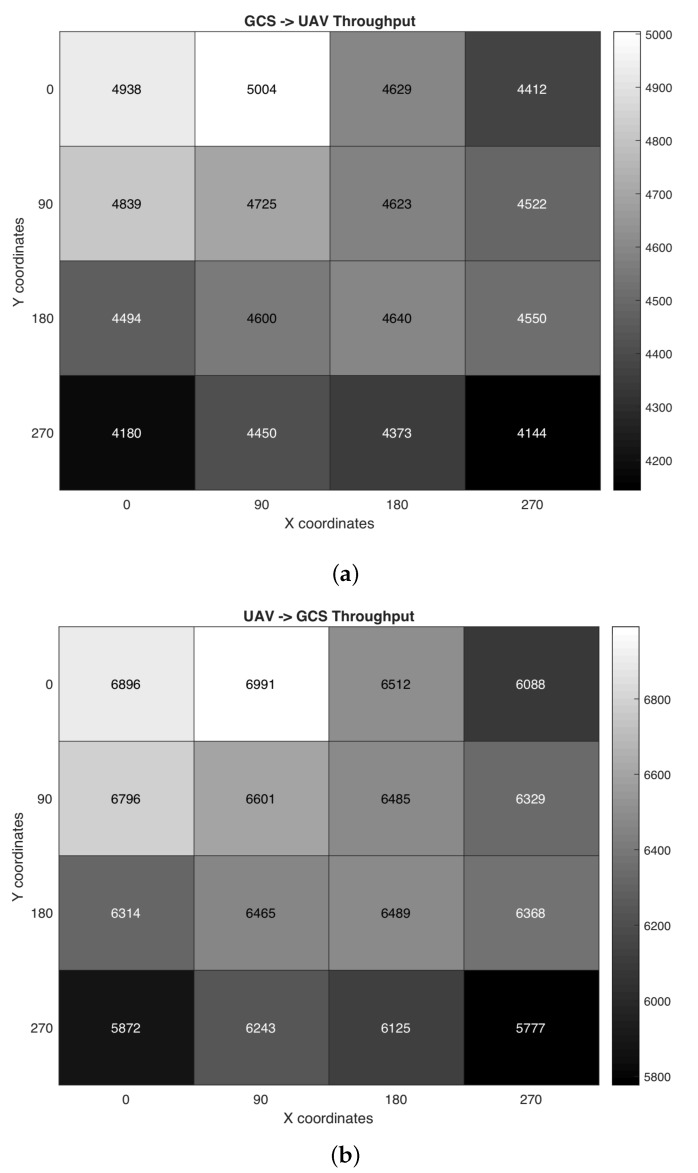
(**a**) GCS-to-UAV orchestration traffic throughput in kbps. (**b**) UAV-to-GCS orchestration traffic throughput in kbps.

To check all the aforementioned issues, we simulated four hours of constant control communications between all the nodes and the GCS, using the battery replacement model described at the beginning of this section. To ensure consistency in our results, we run the simulation 30 times using random values for battery lifetime and replacement times at every run, obtaining the average traffic throughput reception on each node and the GCS depending on the direction of the traffic. The obtained results can be seen in Figure 3.

As it can be observed in the heatmaps, the throughput of control communications is affected in all SUAVs with a various degree of impact depending on the position of the aircraft: when a SUAV is close to the GCS, its throughput is almost the same as if no replacements were performed, nearby the aforementioned 7 and 5 kbps mark, as in SUAVs (1,1) and (1,2), both directly communicating with the GCS. However, the further away we get from the SUAVs directly connected to the station, then the lower the throughput becomes in most cases. This jump in quality is attributed to link breakdowns: when a SUAV is directly linked with the GCS, then its connections do not depend on more aircraft to forward its traffic, which in turn will translate into an almost perfect connection. On the other hand, the longer the distance (in hops) from/to those aircraft is, the more likely it is that routing paths might break, requiring some re-routing and starting the backoff algorithm at the sender until an available path is found again. Hence, we can see that having more hops between a SUAV and the GCS will negatively impact its throughput using TCP because the number of link increases along its traffic path, increasing the chances of a path breakdown and leaving the nodes with more periods where no control communications can be performed between the GCS and the aircraft, only trying to send retransmissions following the instructions of the congestion control algorithm. The best example of this property is the difference between SUAVs (1,2) and (4,4): the throughput decrease is more than 20% in both ways, which is a very noticeable difference against its expected value.

Even though this factor is true in most cases of the topology, it is also possible to spot similar throughput results on SUAVs located at the center of the heatmaps: despite being further away from the aircraft connecting with the GCS, they have very close values with their nearby neighbors, in some cases even slightly improving its performance as in the case of SUAVs (3,2) and (3,3). Moreover, nodes that should have a similar performance with the same number of hops have a wider disparity of values than expected: such is the case of (3,3) and (1,4), where their distance is 2 hops from the nearest link to the GCS (node (1,2)), but their throughput is noticeably worse in the latter case (10% decrease with respect to one another). In all these instances, it can be seen another important property on their connection health: the degree of the nodes affects its throughput as well. Routes in the network will be recomputed each time a SUAV has to be recharged; having more neighbors to forward the TCP traffic aids finding more available routes to reach the destination, while having fewer units will introduce more “dependency” on those surrounding units. The best way to describe this behavior is using an example: SUAV (3,3) has four nodes surrounding it, i.e., a four degree node, so it is more likely to find a route faster (since more paths are available when using its three remaining nodes), and finding all four units simultaneously down due to simultaneous replacements is very unlikely. However, if we choose another aircraft with a lower degree by two nodes, for example SUAV (1,4), then forwarding traffic becomes a far more difficult task for the unit since the possible combinations are fewer, making it easier to find both aircraft offline, preventing the unit from receiving/sending traffic until one of those nodes is back online and the routes converge again. It is easy to spot in this example that, even though they have the same number of hops to a directly connected node to the GCS, SUAV (1,4) has almost 10% less throughput compared to (3,3), which is a noticeable difference in quality. In the worst case, if those nodes cannot be replaced on time before all TCP timers expire, then the aircraft will close the connection until it is replaced again and, in consequence, will close any orchestration communications between the SUAV and GCS until its eventual replacement, severely harm its orchestration efficiency. This is the reason the borders of the network often have worse throughput values compared to those located at the center, despite having the same number of hops to the GCS. Moreover, it is especially noticeable in the topology corners, since those are the ones with the least number of surrounding aircraft, with the worst values of the whole scenario in consequence. This problem could be amplified in other topology types, e.g., a serial topology, since they are connected along a single path: if any aircraft turns off when its battery depletes, then the risk of closing all TCP connection increases if the SUAV is not able to be changed on time, preventing the node from receiving control communications during a long time span and severely harming its performance.

**Figure 4:**
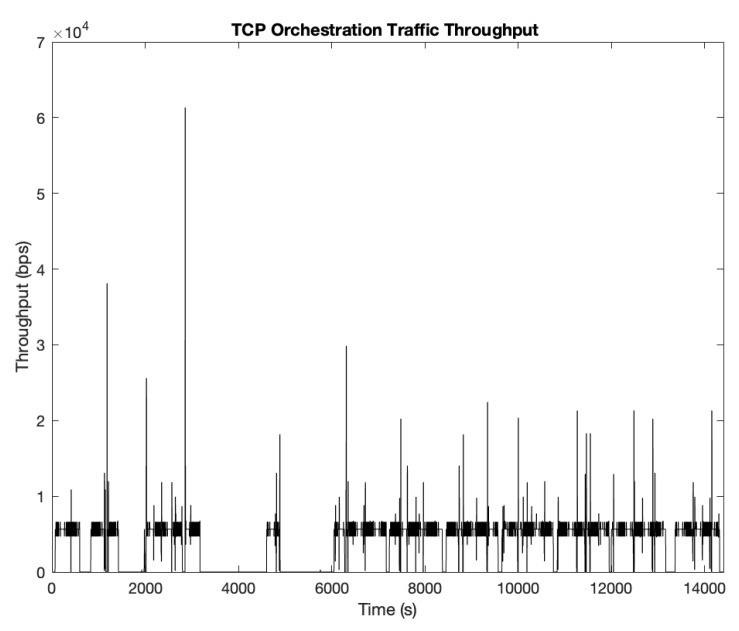
Received throughput on SUAV (4,4) using TCP as transport-layer protocol.

It is easier to spot these reasons when looking at how orchestration traffic evolves in one of the simulation runs. Figure 4 collects the received throughput in the GCS from SUAV (4,4) in one of those simulations, since it is the one that will bring the least favorable results, i.e., where improvements are necessary to increase its performance.

As it can be spotted in Figure 4, there are time periods where its throughput drops throughout the simulation, as OLSR routing needs to converge after one (or several) links are broken due to certain units being replaced. We can see that there are spikes of traffic (traffic bursts suddenly arriving at the node) because TCP sends retransmissions once there is a route recovery after a link has been broken, using the fast-recovery mechanism to send all that traffic directly once it has the opportunity, including previous retransmission received during the breakdown period. This in turn produces a less constant flow of information, and some nodes may receive repeated and outdated information at once when they arrive to the nodel, since its origin are retransmissions from previous queries. However, this is not the most prominent characteristic in this graph: with TCP, there are some portions of time where no orchestration traffic is present at all (around the 4000 s mark and just before reaching the 6000 s one as well). We have previously explained the reasons for this behavior: the unit cannot find a route on time before the TCP backoff algorithm expires, dropping the connection entirely, leaving the nodes without NFV orchestration during that phase until it is replaced at a later moment in time. This is the main source of throughput drops, since there are long periods without any orchestration being performed in the aircraft. This could also introduce other problems with the NFV Orchestrator, since it can interpret this connection unavailability as an issue with the nodes themselves, instead of a connection problem, leading to orchestration inefficiencies because it may think these resources are permanently lost, when in reality is just a temporary situation that will be solved after the UAV replacements and route convergence. These problems could be potentially solved using UDP instead, since the connection will not be interrupted (there is no connection per-se, but rather a source sending traffic to the destination) while also dealing with reliable transmission at the application level in order to avoid resending outdated/useless information since UDP does not guarantee reliability against packet losses.

### 4.2. Orchestration Traffic Performance with Background Data Traffic

In the previous test set, we analyzed the behavior of TCP in an intermittently available SUAV environment in complete isolation from any other traffic inside the network (except OLSR traffic, which is neglectable since it is not very frequent and does not generate much traffic). Nevertheless, typical networks do not behave this way: in all NFV platforms, VNFs will generate traffic to either communicating with other VNFs in the same network or sending traffic outside the network, e.g., sending traffic to the Internet. Hence, this orchestration traffic is not isolated, forced to share the available wireless channel with data communications, which in turn can further affect its performance. With these new test sets we aimed at checking performance for orchestration traffic in a saturation environment using TCP. We performed the same tests with a slight difference: we saturated the network with background TCP traffic to simulate the presence of VNFs exchanging information using the data plane. In this respect, we appended two new traffic sources to our reference scenario: a TCP source attached to SUAV (3,1) sending traffic to SUAV to (2,4), and another one attached to SUAV (1,3) transmitting TCP traffic to (4,3). In both cases, the TCP sources use the maximum transmission rate possible, according to TCP congestion control mechanisms, to analyze the performance of the orchestration traffic under congestion conditions.

**Figure 5:**
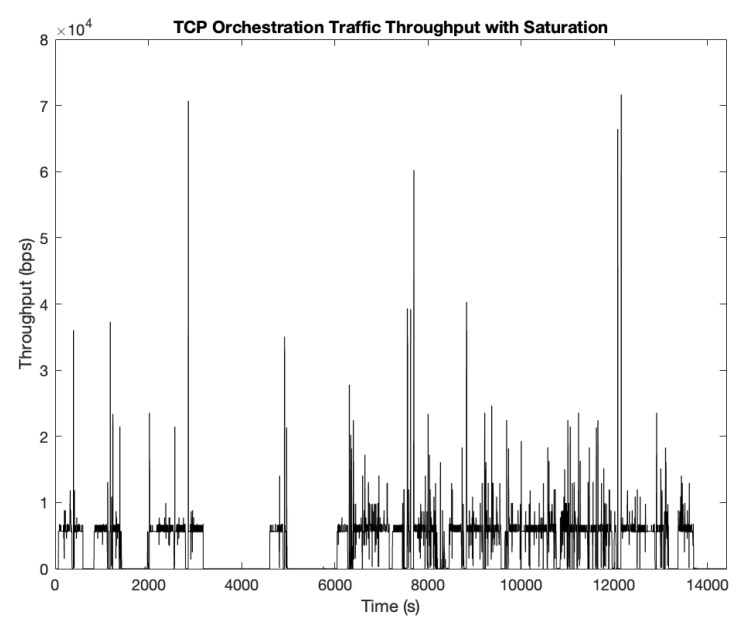
Received throughput on SUAV (4,4) using TCP as transport-layer protocol under saturation conditions.

The results of this experiment can be seen in Figure 5. As it can be observed in this figure, the performance of orchestration traffic clearly decays compared to the idle case: traffic spikes are more frequent and overall throughput is lower. Nevertheless, same periods where no orchestration traffic are still present in the same spots, since the SUAV substitution times did not change. However, there are even more traffic spikes than before because the aircraft only send all of its accumulated traffic (including TCP retransmissions in that case) once it has the opportunity to forward it, which will depend on the TCP connections saturating the network since they are the ones occupying most of the channel bandwidth, resulting in a more irregular throughput distribution with higher traffic bursts, negatively impacting NFV orchestration. The reason of this behavior relies on how the forwarding aircraft deal with orchestration traffic in these conditions: if the transmission buffer of a node in the routing path is full, and either the channel is completely occupied and or a link in the path breaks, it will discard incoming packets since it does not have any more space, dropping those ones that arrive to this node until it has the opportunity to forward that traffic. Hence, more packets will be dropped in the scenario, potentially losing control information useful for the NFV orchestration service. This turns UDP into an attractive solution as an alternative to TCP, whose congestion control mechanisms penalize the performance of orchestration traffic due to the intermittencies present in this medium, both in the absence and presence of background traffic. Nevertheless, the ability to produce retransmissions is still desirable in certain situations, e.g., to avoid missing important orchestration information from the VIM. In any case, we want to highlight that the decision on which information should be delivered reliably, and thus retransmitted if lost, should take place at the application layer (i.e., retransmissions should be implemented as required by the VIM and by the orchestration application services running at the compute nodes). As an example, if the network path between the VIM and a compute node is unavailable for several seconds, the retransmission of information regarding the status of a compute node resources may not be necessary, as the information concerning this status may be outdated once the network path is recovered, and a new application-level request formulated by the VIM may be preferable to get up-to-date status information. In this scenario, where the reliability of data transmission is to be implemented as required by orchestration-related application processes, the unreliable delivery service of UDP may provide a suitable option to disseminate the orchestration information.

### 4.3. Orchestration Traffic Performance Using UDP

**Figure 6:**
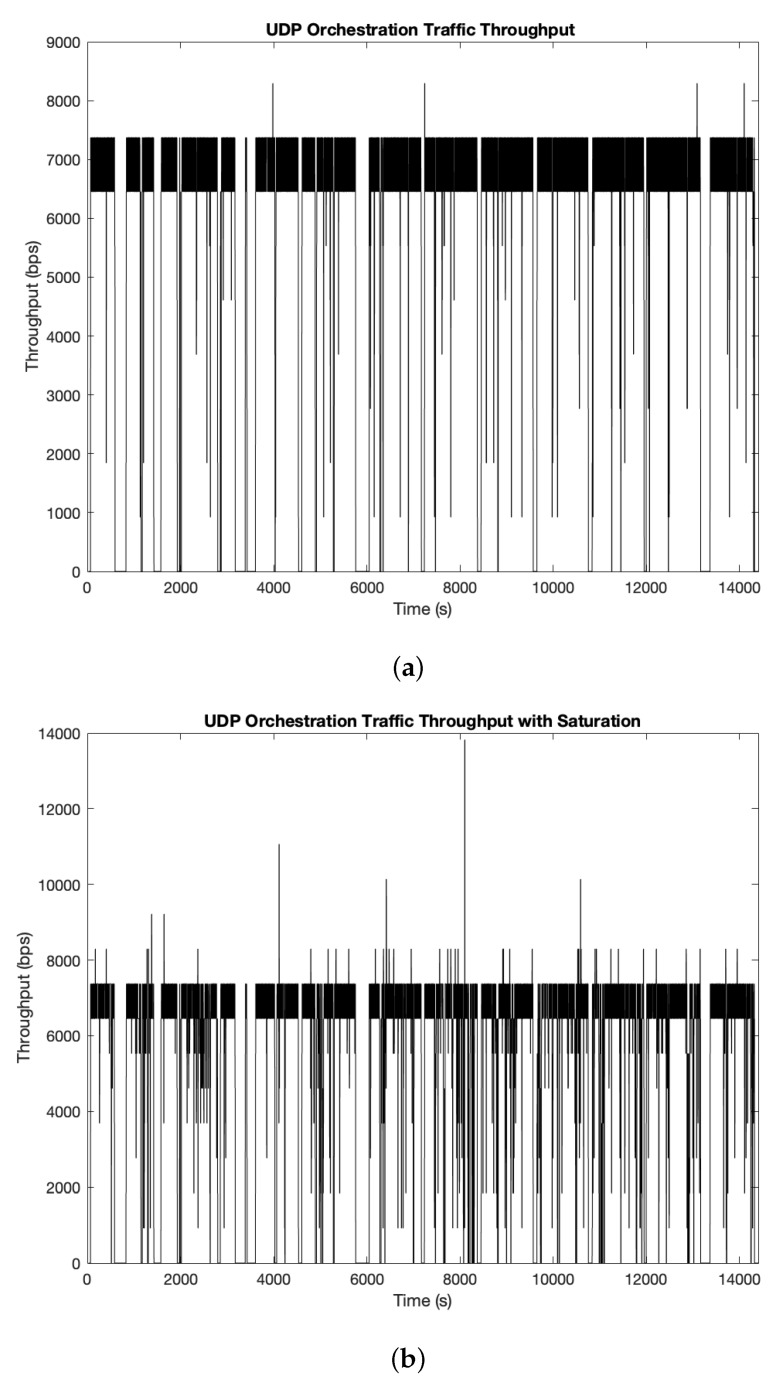
(**a**) Received throughput on SUAV (4,4) using UDP as transport-layer protocol without saturation conditions. (**b**) Received throughput on SUAV (4,4) using UDP as transport-layer protocol under saturation conditions.

In the previous sets we talked about the advantages that using UDP would provide to intermittently available SUAV platforms to disseminate NFV orchestration information. Nonetheless, to explore this possibility, we performed the same sets using UDP under those conditions to see if there is any real advantage against TCP. Figure 6 shows both cases in the same conditions of the previous tests.

As expected, UDP produces a more constant and stable flow of traffic between the aircraft and the GCS as compared to TCP in both cases, more quickly reaching the target rate without being subject to the congestion control mechanisms of TCP. In both cases, there are time periods where the throughput drops throughout the simulation, as OLSR routing needs to converge after several links are broken due to certain units being replaced. As we can see in both situations, in UDP there is no “recovery” per-se (throughput decreases and increases back to its normal rate once the traffic flow is reestablished), so the GCS/SUAV will be receiving packets when there is an available route, no matter how much time passes: as long as the source is generating traffic and a route is available, orchestration traffic will be eventually received, no matter how much time it takes for the network to find an available path. This is completely opposite to TCP, as connections will be dropped if no path is found before their backoff timers expired, leaving the aircraft with periods without control information, severely harming its throughput.

Nonetheless, in the saturation state there is a noticeable worse performance in this case as well. The reason is exactly the same as in the other protocol: if nodes cannot send traffic to the network, either because the channel is occupied or there is no path to the destination, then the units along the routing path will discard the packets arriving if their buffer is full. In contrast with TCP, since there are no retransmissions, the discarded packets are completely lost, which must be taken into account during the orchestration process since important information could be lost until the routing path is available again.

### 4.4. Additional Considerations and Transport-Layer Approaches

After performing the tests and analyzing their results, we still need to consider some additional points that involve these connections and/or the platform itself. First of all, even though control information should have higher priority against traffic data, this should not always be the case: in NFV orchestration, asking about resource state is not the only type of orchestration traffic an NFVO generates: it can produce NFV instantiation deployments, scaling instructions… that will add more traffic to the network, even saturating it at some point (e.g., instantiating VNFs for the first time in a node). Therefore, a smart VIM should be able to balance the competition between orchestration and data traffic, which has to be taken into account when developing a solution based on UDP, since sending a large amount of orchestration traffic through the network could induce packet losses and impact the performance not only of data-plane traffic, but also of orchestration traffic exchanged by other SUAV nodes, harming the performance of the network services deployed over the aerial network and of the orchestration process.

Precisely, this is one of the main disadvantages of using UDP as an alternative to TCP for orchestration traffic: NFV-related control packets could be lost in the delivery process. Since UDP does not have any kind of retransmission mechanism, an alternative way to ensure its reliable delivery is necessary in this environment. In this respect, reliable data transfer could be guaranteed at the application level, either directly by the MANO system or by using a middleware solution. This would allow keeping all advantages from UDP while retaining reliable delivery. Moreover, managing retransmissions of data to ensure reliable transference at application level might be the best solution, since TCP may retransmit outdated information while there is no path to the aircraft/GCS: with an application layer oriented solution however, the retransmitted information could be managed better to ensure providing the most recent information as possible. One example of these solutions could be using the Delay Tolerant Networking (DTN) concept, a promising solution which can spread the control information along the network without being disrupted by replacements, as the information is only forwarded hop-to-hop once a link is available [44]. However, this is a double-edge sword: this information should not remain in the network more time than necessary, since it will be outdated once it reaches its destination if the information remained in transit too much time. Therefore, a solution with this paradigm should consider network latency to avoid spreading outdated information to the NFV nodes.

As we have already mentioned, in the simulations we used the OLSR routing protocol, because it is a well-known and recognized standard, with an existing implementation in NS3, and our prior work indicates that it provides appropriate performance metrics in scenarios as in the one considered in this section. However, it is important to point out that choosing other protocols could impact traffic performance, since additional mechanisms and variables have to be taken into account as well (e.g., route discovery mechanisms, timers, header size, etc.). In a real-life scenario with resource-constrained devices, it would be essential to select a suitable routing protocol that ensures an appropriate performance in the dissemination of management and orchestration information. In this respect, there are several alternatives that could be taken into account to support network routing, such as AODV [34], DSR [33], and AERO [45]. While the first two options have been specifically developed as routing protocols for MANET environments, the latter has been designed to provide route-optimization mechanisms in multi-access links, and could represent a potential solution to support network routing in MANETs with a large number of nodes. In any case, at this point, it is unclear whether the AERO protocol is suited for the resource-constrained ad-hoc scenarios that we are considering in this paper, where the availability of flying nodes may be intermittent.

As a final consideration, in our reference scenario, we assume that an infrastructure provider owns a fleet of SUAVs, each onboarding a general-purpose hardware and software platform (i.e., a compute node in cloud computing terminology). These compute nodes form an aerial cloud computing infrastructure that is under the control of a VIM, which is owned by the infrastructure provider. In addition, compute nodes are interconnected to build a multi-hop wireless ad-hoc network, which supports: (1) the automated provision of telecommunication services over the geographic area where SUAVs operate; and (2) the exchange of orchestration information between the MANO system and the compute nodes, which enables the management of the resources available at compute nodes (i.e., to support the deployment of that telecommunication services over the compute nodes). For simplicity, our reference scenario considers a single GCS, providing a facility with equipment that enables human operators to monitor and control the SUAVs themselves (e.g., transportation of SUAVs to a specific area, execution of pre-flight procedures, control and monitor of taking off/landing procedures, provision of waypoints to control the position of SUAVs, etc.). In this setup, we assume that the VIM runs at a server computer that is available in the GCS, and supports the deployment of virtual functions over the compute nodes transported by the SUAVs.

In any case, it is also possible to divide a large geographical area into smaller regions, and use multiple GCSes (e.g., one per region) to support a larger-scale deployment of SUAVs. In this case, the use of a single VIM from one of the available GCSes is still feasible, as long as this VIM has network connectivity with all the compute nodes onboarded at the SUAV units (i.e., the aerial network built over the large area can support the communication of the VIM with every compute node onboarded by a SUAV). Alternatively, the infrastructure provider could use multiple VIMs, which would be deployed at different GCSes. In this case, the coordination of orchestration actions would be performed by the NFV orchestrator (NFVO), which should have network connectivity with each VIM (e.g., through a satellite data link). In any case (either using a single VIM or multiple VIMs), a VIM controls a set of compute nodes attached to SUAV units, a situation that is appropriately represented by our reference scenario.

## 5. Conclusions

Management and orchestration of infrastructure resources and virtual functions are fundamental to coordinate the operation of NFV environments. The proliferation of new enabling technologies in the telecommunications market, such as SUAVs, creates new opportunities for the fast and cost-effective deployment of network services following the novel NFV paradigm. However, this opportunity opens new challenges and hurdles to NFV orchestration: (1) the limited lifetime of NVFI nodes, which has to be taken into account for migrating nodes and functions in replacement cases; (2) the intermittent availability of control communications, which can make nodes unavailable for communications in short periods of time; (3) the limited capacity of NFVI nodes, which affects the load an SUAV is able to carry and the protocol used for exchanging information; (4) the transport-layer protocols for control communications, whose performance may be decreased in mobile ad-hoc networks, as in the case of TCP; and (5) the support of enhanced policies for VNF placement, which current VIMs do not provide as they are not aware of SUAV specific aspects, such as battery constraints and geographic location [6].

In this paper, we performed a set of simulations to check the behavior of orchestration traffic when TCP is used as the underlaying transport-level protocol. To do so, we defined some tests with a set of SUAVs forming a grid topology where aircraft had to be replaced after some period of time, simulating some battery depletion and subsequent replacement. Afterwards, we took a look at the average throughput to check how intermittencies affected the orchestration traffic performance. Our results show that the number of hops a SUAV is from the GCS has a direct impact in the throughput of the orchestration traffic exchanged with the SUAV, since the longer the path is the more likely that routing paths will be broken. However, this is not the only reason that may cause performance degradation. The number of neighbors of a SUAV in the aerial network (that is, the SUAV degree) also correlates with the performance of orchestration traffic, since having less nodes around an aircraft could entirely sever a TCP connection if the routing protocol does not converge before the backoff timers from TCP expire. This is a problematic issue that needs to be considered by MANO platform implementations operating on network environments such as those considered in this paper. Moreover, under close to congestion conditions this effect is much more pronounced, and UDP is able to provide a more stable throughput as compared to TCP, enabling more time periods where the SUAV nodes and the GCS receive the control, while TCP has more idle periods that hurdle NFV orchestration actions over the aerial network.

Our future work includes the implementation of our SUAV reference scenario. For this purpose, we will use an emulation platform for multi-UAV applications that we are developing [46]. In addition, we will continue developing solutions to address the rest of the challenges to NFV orchestration over resource-constrained environments that we have identified in this paper.

## Figures and Tables

**Table 1:** Challenges of NFV orchestration in Resource-Constrained Aerial Networks.

Object of Analysis	Challenges/Hurdles	Potential Approaches
Limited lifetime of NFVI nodes	SUAVs are autonomous devices that require some power supply source to be operative, and performing different actions such as traffic relaying or executing software functions will eventually deplete their battery lifetime. All VNFs running inside its payload must be migrated into another operative unit to avoid, or mitigate, operational cuts on the services being provided. There are no known VIM implementations that consider battery lifetime as a limited resource, making VNF migration in these environments suboptimal.	Develop a VIM able to consider battery lifetime for scheduling VNF migration in advance to increase NFV management and orchestration performance using planning algorithms [23]. Perch some SUAVs on land in specific-purpose ground structures. Use different batteries for flight management and computational resources
Intermittent availability of control communications	Battery depletion turns these units into volatile nodes that must be replaced by other units. These disconnections will break links between the all SUAVs and the GCS, potentially leaving some nodes unavailable to the MANO system. This disruption will be mostly transient however, as either the routing protocols will likely find a new path, and/or the units are replaced after some time have passed to converge into a stable state. It is important for a VIM to take into account that this transient state is the normal behavior on the aircraft, and not interpret these failures as permanent. The inclusion of mobility and other wireless phenomena (fade-away, weather, etc.) could increase this problem since links breakdowns will be more frequent.	Develop a VIM able to take into account the intermittent availability of control communications by supporting a reasonable delay for management and orchestration actions/information retrieval for the NFVI nodes.
Limited capacity of NFVI nodes	The reduced size of SUAVs constrain the available resources for their payload, including its weigh, size and processing power. Most commercial VIMs use HTTP as its default application-layer protocol, which is not the most appropriate choice for resource-constrained environments, as it could be considered a process-intensive protocol for this units due to the amount of processing power required for its usage.	Reduce the necessary resources to support NFV by using techniques such as lightweight VNFs or paravirtualization. Use lightweight protocols/IoT such as CoAP, QUIC or MQTT to reduce message overhead for a more cost-effective solution to save battery lifetime and reduce the computational resources needed.
Transport-layer protocols for control communications	On its conception, TCP was developed as a protocol for reliable data transfer over wired networks. In most cases, link-related error would rarely occur, and most packet losses would be related to congestion. However, wireless technologies completely changed how communications could be done, but introduced new challenges that increased link-related failures due to several factors such as the instability of the medium and mobility. TCP was never intended to deal with temporary and/or common link failures, which could potentially affect its use in wireless environments, creating new drawbacks that had to be dealt with in several layers such as the physical and network layers. In summary, TCP is not able to recognize the source of the failure in wireless media, having to use the congestion control mechanism when there is no congestion in the network, decreasing the overall throughput that can harm its management and orchestration performance because its traffic is not high in comparison with the available bandwidth. Even though TCP might not be the most suitable protocol for this exchange, there are some characteristics we want to preserve such as reliable data transference or retransmission policies.	Use protocols that rely on UDP as its main transport-layer protocol, while moving its reliability mechanisms to the application layer. Examples of this solutions could be: 1) CoAP, which applies the HTTP REST model to be available in resource-constrained environments. 2) QUIC, which is a new transport-layer protocol to imitate an implementation of HTTPv2 + TSL using UDP.
Enhanced policies for VNF placement	VIMs do not take into account parameters such as battery lifetime or geographical positions when allocating VNFs on NFVI nodes.	An specialized VIM could take into account battery lifetime to assign critical VNFs in healthier nodes. Moreover, orchestration could also be improved by using this parameter, as knowing the remaining capacity is essential to trigger VNF migration as well as re-allocation policies. To manage the placement of SUAVs in a geographical area, implement the flight control engine as a VNF on every unit, providing its flight trajectories using a VNFM as configuration parameters, which could be used to specify their movement at the deployment of the NS.

**Table 2:** This is a table caption. Tables should be placed in the main text near to the first time they are cited.

Simulation Parameters	Values
Number of Nodes	15
WiFi Range	70 m
Simulation Time	14,430 s
Modulation Scheme	OFDM at 6 Mbps
